# A Systematic Review on Drugs Acting as Nicotinic Acetylcholine Receptor Agonists in the Treatment of Dementia

**DOI:** 10.3390/cells13030237

**Published:** 2024-01-26

**Authors:** Alessio Crestini, Elena Carbone, Roberto Rivabene, Antonio Ancidoni, Paolo Rosa, Ada Maria Tata, Elisa Fabrizi, Nicoletta Locuratolo, Nicola Vanacore, Eleonora Lacorte, Paola Piscopo

**Affiliations:** 1 Department of Neuroscience, Italian National Institute of Health, 00161 Rome, Italy; elenacarbone96@gmail.com (E.C.); roberto.rivabene@iss.it (R.R.); paola.piscopo@iss.it (P.P.); 2 National Center for Disease Prevention and Health Promotion, Italian National Institute of Health, 00161 Rome, Italy; antonio.ancidoni@iss.it (A.A.); nicoletta.locuratolo@iss.it (N.L.); nicola.vanacore@iss.it (N.V.); eleonora.lacorte@iss.it (E.L.); 3 Department of Medical-Surgical Sciences and Biotechnologies, Sapienza University of Rome, Polo Pontino, 04100 Latina, Italy; p.rosa@uniroma1.it; 4 ICOT (Institute of Traumatology and Orthopaedic Surgery), 04100 Latina, Italy; 5 Department of Biology and Biotechnologies Charles Darwin, Sapienza University of Rome, 00185 Rome, Italy; adamaria.tata@uniroma1.it; 6 Research Center in Neurobiology Daniel Bovet, Sapienza University of Rome, 00185 Rome, Italy; 7 Doctoral School, The Catholic University of Valencia San Vicente Mártir, 46001 Valencia, Spain

**Keywords:** nicotinic acetylcholine receptor, nicotinic agonists, dementia, Alzheimer’s disease, neurodegenerative disease, disease-modifying therapies, clinical trials

## Abstract

Acetylcholine signaling is attenuated in early Alzheimer’s disease (AD) and other dementias. A significant reduction in the expression of nicotinic acetylcholine receptors (nAChRs) in the brain of AD patients has also been reported in several molecular biological and in situ labeling studies. The modulation of the functional deficit of the cholinergic system as a pharmacological target could therefore have a clinical benefit, which is not to be neglected. This systematic review was conducted to identify clinical trials, which evaluated the safety and efficacy of nicotinic acetylcholine receptor agonists using Clinicaltrial (CT) and EudraCT databases. Structured searches identified 39 trials, which used 15 different drugs designed to increase the function of the nAChRs. Most of the identified clinical trials were phase II trials, with some of them classified as ongoing for several years. The systematic screening of the literature led to the selection of 14 studies out of the 8261 bibliographic records retrieved. Six trials reported detailed data on adverse events associated with the intervention, while twelve trials reported data on efficacy measures, such as attention, behavior and cognition. Overall, smost of the physical side effects of cholinergic agonists were reported to be well tolerated. Some trials also reported improvements in attention. However, the efficacy of these drugs in other cognitive and behavioral outcomes remains highly controversial.

## 1. Introduction

In recent years, an increase in the frequency of neurodegenerative diseases has been observed, with Alzheimer’s disease (AD)—the most common neurodegenerative form of dementia—affecting about 17% of the population around the age of 75 [1]. Estimates suggest that in Europe and worldwide, cases will be, respectively, more than two times and three times higher by 2050 [2]. AD, from its earliest stages, is characterized by the progressive formation of aggregates in brain tissue, such as senile plaques and neurofibrillary tangles, which can cause neuronal degeneration and death, resulting in severe memory loss, cognitive impairment, language difficulties [3] and behavioral and personality changes. Partially due to a still limited understanding of the mechanisms underlying the pathogenesis of AD, the degenerative process and trajectory remain unmodifiable, and attempts at identifying disease-modifying pharmacological interventions capable of significantly improving the cognitive outcomes have not been particularly effective. This is the case with antibodies directed against β-amyloid oligomers, which, despite the amyloid cascade hypothesis, have encountered major difficulties in obtaining regulatory approval for their marketing. However, in the absence of significantly effective treatments for dementia, reconsidering other etiopathological hypotheses remains necessary, which can explain and help target the underlying degenerative processes. In this regard, another widely accepted hypothesis for explaining the etiology of AD in its early stages is related to the cholinergic system [4,5]. The only currently established symptomatic treatment for AD involves cholinesterase inhibitors, which reduce the hydrolysis of acetylcholine in the synapses of cholinergic neurons [6].

### 1.1. Nicotinic Acetylcholine Receptors in CNS

Acetylcholine receptors (AChRs) are another functional element of the cholinergic system, which has been extensively studied in biomedical research as a potential therapeutic target in AD. In particular, nicotinic acetylcholine receptors (nAChRs) are ligand-gated ion channels, which are widely distributed in most brain regions [7,8]. nAChRs are expressed by neurons at both the pre-synaptic and post-synaptic levels, and they affect several physiological and behavioral processes by regulating neuronal excitability and neurotransmitter release [9]. Neuronal nAChRs are composed of α and β subunits, which assemble to form either a heteromeric or a homomeric configuration [10]. Nine α subunits (α2–α7, α9 and α10) and three β subunits (β2–β4) have been identified in brain tissue [11]. The two major subtypes of nAChRs in mammalian brain are the α7 and α4β2, which are also the subtypes most commonly involved in neurological disorders, including AD [12]. The distribution of nAChRs is well preserved across vertebrate species. Both the α4β2 and α7 subtypes show a broad distribution throughout the brain (with overlapping expression areas). A higher density of α4β2 nAChRs is found in the basal forebrain (nucleus basalis of Meynert) and thalamus areas, while moderate expression is observed in the putamen and cerebellum. The lowest levels of α4β2 nAChRs are found in the cortical regions [13,14,15]. α7 nAChRs are sparsely present in the cortex, while their expression is higher in the hippocampus, particularly in the CA1–CA3 and dentate gyrus regions, the thalamus and the basal forebrain [16,17]. The nAChRs expressed in these areas have been proven to be involved in controlling excitability, transmitter release, synaptic function and plasticity, learning, memory, arousal and attention [18].

### 1.2. Nicotinic Acetylcholine Receptors in Dementia

According to their expression patterns and the brain mechanisms they regulate, the functional activity of nicotinic receptors is involved in cognition and the pathophysiology of several neurological and psychiatric disorders, including primary dementias, such as AD and Parkinson’s disease (PD) [12]. An impairment of nicotinic transmission has been observed in pathological condition affecting cortical neurons in AD, both in terms of binding site reduction and a shift in the levels of subunits contributing to receptor composition [19], whereas no significant alterations in the muscarinic acetylcholine receptor expression were observed even in the most advanced stages of AD [20]. Indeed, cognitive impairment has been directly correlated with α4β2-nAChR availability [21,22]. Furthermore, considering the pathogenic mechanisms associated with AD, the same Aβ1-42 oligomers, which accumulate in brain tissue, could be a direct contributor to cholinergic hypofunction. This toxic peptide binds with high affinity to homologous α7nAChR and heteromeric α7β2 nAChR [23], forming Aβ1-42–α7nAChR complexes in those areas, which are most directly linked to memory and cognition [24,25]. The functional consequences of these complex formations include the removal of cholinergic receptors from the plasma membrane of neurons through endocytosis [26], resulting in its potentially neurotoxic intracellular accumulation, the internalization of Aβ1-42 peptides in an environment, which favors their aggregation, and finally, functional impairment of the cholinergic system [27]. Moreover, neuroinflammation, an important risk factor for AD and non-AD dementia [28], could be modulated by the loss of the nicotinic phenotype, mainly related to α7 nAChRs expressed in neuronal and non-neuronal cells, which mediate the cholinergic anti-inflammatory pathway (CAIP) [29]. Growing evidence suggests that CAIP stimulation could modulate microglial activation and reduce the release of pro-inflammatory cytokines in the brain parenchyma [30,31,32]. Overall, several bodies of evidence point to a primary pathological role of nicotinic receptors in dementia, providing a solid theoretical basis for the development of disease-modifying therapeutic approaches linked to the restoration of nAChRs function. The aim of this systematic review (SR) was to provide an overview of all published and unpublished data of all clinical trials investigating the safety and efficacy of drugs targeting nAChRs in participants with dementia.

## 2. Materials and Methods

### 2.1. Methods

This SR was carried out based on the methodology published [33] and reported following the PRISMA statement for reporting systematic reviews and meta-analyses [34]. 

### 2.2. Search Strategy and Selection Criteria

The first structured search was performed on registration databases ClinicalTrials.gov (CT) and the European Clinical Trials Register (EuCT) using the following search terms: (Alzheimer OR dementia OR MCI). The results were not limited based on status, study design, study phase, date of publication or language. Only studies investigating pharmacological compounds specifically targeting nicotinic receptors and enrolling participants with a diagnosis of any type of primary dementia or MCI were considered. The resulting list of registered trials was further analyzed to identify all trials with available results. Based on the list of molecules obtained from CT and EuCT, a specific structured search string was defined and adopted to perform bibliographic searches on databases PubMed, Embase and the Cochrane Library. The following terms were used: (dementia* OR Alzheimer* OR “mild cognitive impairment” OR MCI) AND (nicotin* OR Vareniclin* OR Champix OR GTS-21 OR DMXB-A OR DNBX-anabasein* OR EVP-6124 OR MT-4666 OR Enceniclin* OR ABT-089 OR Pozaniclin* OR ABT-126 OR Neloniclin* OR EVP-6124 OR AZD3480 OR Isproniclin* OR TC-1734 OR AZD-1480 OR SSR180711 OR Dimebon* OR Dimebolin* OR Latrepirdin* OR Pf-01913539 OR Simufilam* OR PTI-125, sumifilam* OR Nefiracetam* OR DM-9384 OR RO5313534 OR RG3487 OR MEM 3454). All literature up to May 31, 2023 was included. No limitations were applied in relation to the publication date, study design and language. Based on the results from both searches, a list of all trials with available results from any of the considered sources was defined, and the source of data was specified.

### 2.3. Study Selection

After removing duplicates, the list of references resulting from bibliographic searches was uploaded on the online tool Rayyan (https://www.rayyan.ai/, (accessed on 1 December 2023)), and abstracts were selected based on their pertinence and relevance to the topic of the review. The full texts of selected studies were retrieved, and the pre-defined eligibility criteria were applied. The references of the included studies were also analyzed to further identify potentially relevant publications. The following eligibility criteria were applied: all trials reporting data on the safety and efficacy of any type of drug specifically targeting nicotinic receptors for the treatment of people with a diagnosis of any type of dementia or MCI at any stage were included. Studies enrolling only healthy participants or subjects with any condition other than MCI or dementia were excluded. Letters, commentaries, editorials, conference proceedings, case reports or case series, and non-systematic and narrative reviews were also excluded. Systematic reviews, where available, were considered separately to check for references and consistency of results. Literature selection, data extraction and qualitative assessment were all performed by four independent reviewers (PP, EC, AC, RR). Disagreements, where present, were resolved by consensus or by referring to an independent reviewer. 

### 2.4. Data Extraction and Quality Assessment

After defining the list of all registered studies, the available results from each registered trial were searched in both published studies and registration databases (i.e., CT and EuCT). For all trials for which data were available, the source of information was recorded (i.e., unpublished results retrieved from registration databases and published studies). Data extraction was performed using standardized forms. The gathered data included the year of publication, characteristics of the included population, type of drug and results for all considered outcomes. In the case of trials with information available from both sources, data were compared, and if any inconsistencies were present, the most recent source was considered as valid. Supplementary materials from included studies were also taken into consideration where available. Only published RCTs were qualitatively assessed using the Cochrane Risk of Bias (RoB) tool [33], as too much information on methodology was missing from the results available from the registration platforms. Quality assessment was performed using the RevMan software version 5.4 and was reported in tabular form. According to the RoB tool, the risk of bias was classified as “Low”, “High” or “Unclear”, and other potential biases and/or methodological flaws or limitations were also considered.

### 2.5. Data Synthesis and Analysis

The results were summarized in both tabular and narrative form. A quantitative analysis of data through meta-analyses was not performed mainly due to the heterogeneity of the considered interventions. With regard to safety outcomes, data on the type and frequency of any type of adverse events (AEs) and treatment-related adverse events (TEAEs) were gathered, along with data on the type and frequency of serious adverse events (SAEs) and any other type of safety-related outcomes reported in the included studies. In relation to efficacy outcomes, data on the mean change from baseline of the MMSE, ADAS-Cog or CDR-SB scales or any other clinical cognitive scales were gathered. Moreover, data on PET, fMRI or any other considered neuroimaging measures, along with any other type of efficacy measure, were also included.

## 3. Results

Overall, 4295 records were retrieved through searches on CT and EuCT. After screening and removing duplicates, 39 trial protocols investigating drugs targeted at nicotinic receptors in participants with AD or dementia were included (Table 1).

**Figure 1:**
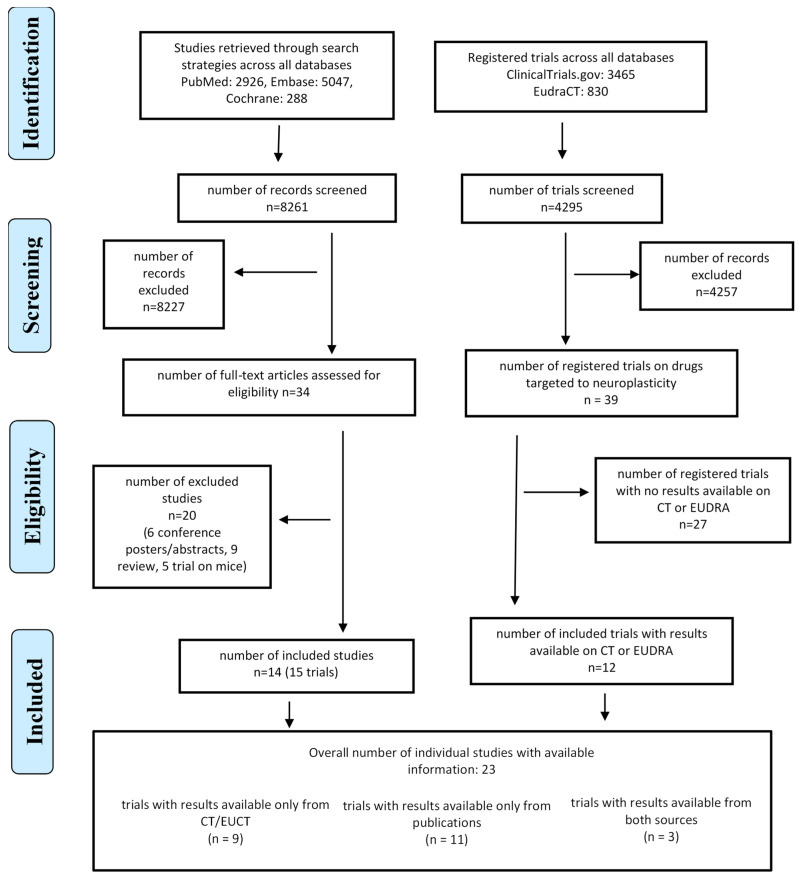
Modified PRISMA flow diagram for clinical trial selection.

The literature searches in bibliographic databases yielded 8261 records, of which 8227 were excluded after the first screening. A total of 34 studies were retrieved in full text and assessed for inclusion. After applying the pre-defined eligibility criteria, 20 studies were further excluded, while 14 publications met the eligibility criteria. The flow diagram of the included studies is reported in Figure 1. 

Among the identified trials, 2 of them were classified as phase I RCT, 30 as phase II RCT, 6 as phase III RCT, and 1 did not report information on the phase. Only 18 trials were reported as completed. Data were available for 23 trials, with 3 having data available from both CT/EuCT and published studies, 11 only from published articles, 6 from EuCT and 3 only from CT (Table 2).

The 39 registered trials investigated 16 different drugs, but the 14 studies for which data were available were carried out on only 7 drugs: nicotine, ABT-418, varenicline, ABT-089, ABT-126, AZD3480, PTI-125.

### 3.1. Methodological Quality of Published Studies

**Figure 2:**
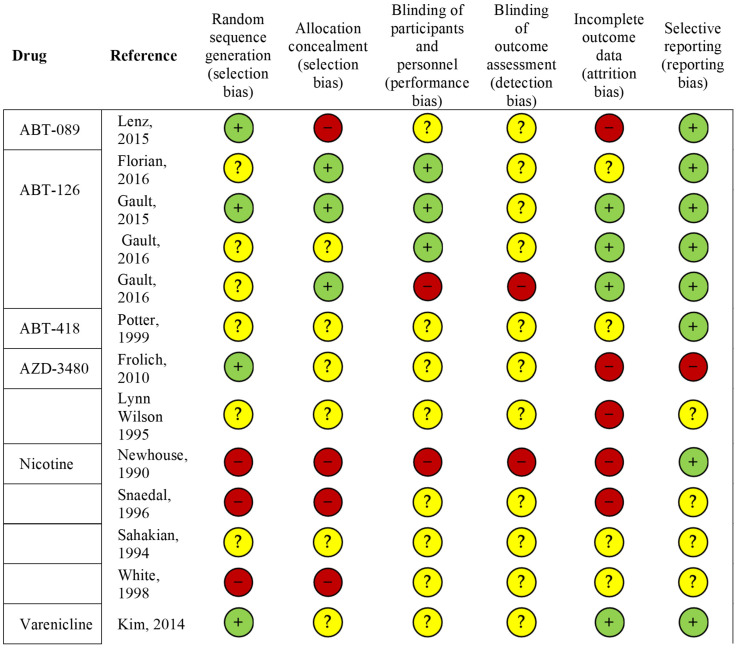
Risk of bias in the clinical trial studies included (*n* = 13). Summary of scores for each domain for each included study. The symbols “+ “, “-” and “?” indicate low, high and unclear risk of bias, respectively. References: Lenz, 2015 [42]; Florian, 2016 [43]; Gault, 2015 [45]; Gault, 2016 [46]; Potter, 1999 [39], Frolich, 2010 [47], Lynn Wilson, 1995 [48]; Newhouse, 1990 [35]; Snaedal, 1996 [36]; Sahakian, 1994 [37]; White, 1998 [38]; Kim, 2014 [41].

Assessment of the methodological quality was performed only for studies published in journal articles (Figure 2). The overall quality of the included studies was moderate to high. The main observed limitations included a lack of information on how the randomization process was conducted and incomplete data on the procedures for allocation concealment and blinding of outcome assessment. Moreover, most of the studies had limited sample sizes, such as one RCT on ABT-418 including 6 participants [39], and were considered by the authors themselves as pilot studies. Moreover, some of the included studies did not report data on the characteristics of participants who were lost to follow-up, thereby preventing the assessment of whether participants who were lost to follow-up or who withdrew from the study were a selected population (e.g., older participants, more severe cases, etc.). 

Another potential bias was found in some studies characterized by a within-subject cross-over design, in which patients who started on a placebo were reallocated to active treatment after 2, 3 or 4 weeks of washout [36,37,38,41] and vice versa. Treated and placebo subjects were merged as AD placebo or AD drug groups without considering the different study designs.

### 3.2. Drug Mechanisms of Action

Among the considered molecules, nicotine belongs to a family of compounds known as alkaloids, which are found in the tobacco plant [49]. Nicotine is an agonist of nAChrs, which includes five α or β subunits, which are found both in the central (CNS) and peripheral nervous system (PNS) [50,51]. According to the cholinergic hypothesis, the cognitive decline in AD results from a lack of central cholinergic neurotransmission due to the loss of acetylcholine [52]. Changes in the expression and density of α7 nAChRs in the hippocampus have also been observed in AD and appear to have the most impact on cognitive function [53]. Therefore, agonists of α7 nAChRs, including nicotine, may be useful for treating AD, as the stimulation of nAChRs in the CNS regulates the release of different neurotransmitters, such as dopamine, glutamate, serotonin, norepinephrine and γ-aminobutyric acid [49]. Nicotine’s stimulation of nAChRs likely affects downstream signaling molecules, including protein kinases, which are important regulators of synaptic plasticity and memory [54]. In particular, protein kinase B is a molecule of the phosphoinositide 3-kinase (PI3K)/Akt signaling pathway, which plays a relevant role in the regulatory functions of neurons in the CNS, including neuronal survival [55,56,57] and learning and memory encoding [57,58,59]. Therefore, the stimulation of nAChRs by nicotine or its analogs is hypothesized to activate the PI3K/Akt signaling pathway, which regulates the learning and memory processes [56,60], and acute and chronic administration of nicotine was reported to improve cognitive impairment in a rat model of AD [61]. Several studies suggest that the addictive effects of nicotine occur through interaction with its receptors in the mesolimbic dopamine system, particularly ventral tegmental area neurons, where nAChRs act to promote the release of dopamine. Chronic treatment with nicotine in fact leads to an upregulation in the number of α4β2-subunit nAChRs. Moreover, N-methyl-D-aspartate and gamma-aminobutyric acid receptors are also involved in the regulation of dopamine release [62]. Varenicline has shown a high affinity for α4β2 nAChRs; it also seems to be a high-affinity partial agonist in α6β2-containing (α6β2*) nAChRs [63]. Activation of α4β2 nAChRs in the ventral tegmental area triggers downstream events, such as increased mesolimbic dopamine release to approximately 50% of the maximum effect of nicotine, which transmits salient reward and opposed signals to higher cortical centers. Recent studies showed that recurrent use of nicotine can cause repeated rapid and transitory increases in dopamine release, which facilitates association and learning but also leads to addiction [64,65,66]. However, varenicline stimulates the basal mesolimbic dopamine release, inhibits nicotine-induced dopamine release and reduces nicotine self-administration, and for these reasons, it is used as a drug treatment for smoking cessation [66,67]. 

ABT-089, 2-methyl-3-(2-(S)-pyrrolidinylmethoxy)pyridine dihydrochloride salt, is a selective neuronal nicotinic receptor (NNR) modulator with enhancing properties for cognitive functions in animal models [68]. It is also known as pozanicline and has shown selectivity and high affinity for the α4β2 subtype in both rat and human NNRs [69]. ABT-089 has different activities, including being an agonist, partial agonist and antagonist, depending on the NNR subtype [69,70]. Decker et al. showed that ABT-089 enhances performance in delayed match-to-sample in monkeys and Morris water maze in rats with deficit induced by surgical or pharmacologic lesion [71], while Sullivan et al. showed that ABT-089 acts as an α4β2-nAChR partial agonist to stimulate [3H]-dopamine ([3H]DA) release in rat striatal slices [69]. 

AZD3480 is another NNR, also known as TC-1734 or isopronicline, (S-E-[4-(5-Isopropoxy-pyr- idin-3-yl)-1-methyl-but-3-enyl]-methyl-amine, and it is a nicotinic receptor agonist with a high affinity and selectivity for the α4β2 and low affinity for CNS α7 receptors. Initially, the neuroprotective effects of TC-1734 were assessed using glutamate toxicity in mature cultures of rat forebrain neurons [72]; then, its enhancing effects on memory were investigated in mice and rats using standard animal models of learning, memory, and its neuroprotective properties were investigated in various animal models. The drug did not show any addictive-like behaviors and had a low toxicity profile [73,74]. Similar but less significant effects were observed with nicotine. Therefore, AZD3480 appears to be a potential candidate for AD treatment and other cognitive disorders in the elderly. ABT-126 ((1R,4R,5S)-4-(5-Phenyl-[1,3,4]thiadiazol-2-yloxy)-1-aza-tricyclo[3.3.1.13,7]), also known as decane or nelonicline, is an α7 nAChR partial agonist. It has a high binding affinity for α7 nAChR, with a 74% maximum agonist activity [75]. ABT-126 showed pro-cognitive effects as a monotherapy in the treatment of AD [43]. 

ABT-418, [(S)-3-methyl-5-(1-methyl-2-pyrrolidinyl)-isoxazole)], is a novel bioisostere of nicotine with highly selective binding to central nicotinic receptors but with no significant activity in dopamine, serotonin, muscarinic, GABA or other G-protein-linked receptors or ligand-gated ion channels, which showed high affinity for central α4β2 [76]. Instead, it showed selectivity for the [3H]cytisine-labeled nicotine-binding site but was minimally active at the neuromuscular junction or α-bungarotoxin-sensitive nicotinic receptor in vitro. ABT-418 has greater selectivity for the α4β2 nicotinic receptor subtype, and it produces some in vitro effects, which are similar to those produced by nicotine [77]. In animal studies, it was shown to exert similar effects as nicotine on behavior, locomotor activity and learning, but with a considerably larger therapeutic index and generally more robust effects on learning and memory, as well as anxiolytic effects [76,78].

PTI-125, also known as simufilam, binds the scaffolding protein filamin A (FLNA), a ubiquitous scaffolding protein and regulator of the actin cytoskeleton [79]. Aβ1-42 signaling via α7nAChR requires the association of FLNA with α7nAChR; therefore, by binding FLNA, PTI-125 reduces Aβ1-42’s binding affinity for α7nAChR, thereby preventing Aβ1-42’s signaling and further accumulation on α7nAChRs [80]. Wang’s pre-clinical study showed that PTI-125 can prevent and reverse the binding of Aβ1-42 to α7nAChR. Concomitant intraperitoneal PTI-125 injections prevented this association, reduced tau phosphorylation and amyloid deposition, and normalized signaling through the α7, NDMA and insulin receptors. The study claimed that Aβ1-42 induced a conformational change in filamin, which would promote its association with the α7 and Toll-like receptors, enabling Aβ1-42 toxicity and inflammation. PTI-125 was said to preferentially bind altered filamin and normalize its conformation [44].

### 3.3. Safety

Overall, all drugs considered in this SR were reported as substantially well tolerated. Therefore, the data on safety, tolerability and frequency of AEs and SAEs were not consistently reported. Only two studies out of the six reporting data on nicotine [36,40] reported the presence of AEs, with the majority being mild and considered as unrelated to the treatment. However, the information on whether these AEs were dose-dependent or not was not reported. No SAEs were mentioned. The only study investigating ABT-089 [42] reported an AE incidence of 59.2% in the ABT group and 60.4% in the placebo group, while the incidence of SAEs was 5.9% in the ABT group and 6% in the placebo group. Across all doses of ABT-089, ranging from 5 to 35 mg over 12 weeks of treatment, the safety profile was similar between groups. Nausea was the only treatment-related AE for which a statistically significant difference between groups was observed, with lower frequency observed in the ABT-089 group compared to the placebo group (*p* = 0.028). With regard to studies investigating ABT-126, one study reported that the drug (25 and 75 mg, 24 weeks of treatment) was well tolerated, and only minor AEs were observed both in the treatment and placebo arms, with comparable overall incidence (67.7% and 67.8%, respectively) [43]. No significant differences in SAEs were reported across treatment groups, with an incidence of 6.3% in the 25 mg ABT-126 group, 6.9% in the 75 mg ABT-126 group and 8.9% in the placebo group. No deaths were reported. Another study (5 and 25 mg, 12 weeks of treatment) reported no statistically significant differences between groups in the incidence of AEs, TEAEs and in the number of participants who experienced AEs (incidence of 40.9%), with the overall severity being mild to moderate [45]. All SAEs were considered as not related to the study drug. One further study (25, 50 and 75 mg, 12 weeks of treatment) reported no differences between groups in the frequency of AEs, with most AEs (95.5%) being categorized by the investigator as mild or moderate in severity [46]. Twenty-seven subjects (6.2%) discontinued their participation in the study prematurely due to AEs. However, the frequency of reported SAEs was comparable across the treatment groups (5.3%). Two deaths occurred during the study, both in the donepezil group, which was included in the study to allow for a comparison of the effects of ABT with an active control. However, both events were considered as not related to the drug treatment. In relation to the study investigating ABT-418 (6, 12 and 23 mg, 4 days of treatment) [39], no data on either AEs or SAEs were reported, while the only study on AZT3480 (5, 20, 35 and 100 mg, 12 weeks of treatment) [47] reported no differences in the incidence of any AE between the ABT group (34.9%) and the placebo group (36.6%). Conversely, a slightly higher frequency of AEs was observed in participants treated with donepezil (37.5%) compared to placebo. No SAEs or deaths were reported. The only study investigating varenicline (1 mg, 12 weeks of treatment) [41] reported a higher frequency of TEAEs in the treatment group (93.3%) compared to the placebo group (62.7%). Two subjects reported treatment-emergent SAEs in the varenicline group, while no SAEs were reported in the placebo group. The only study investigating PTI-125 (100 mg, 28 days of treatment) [44] reported that the drug was safe and well tolerated, with no drug-related AEs/SAEs observed during the study. 

### 3.4. Efficacy

Overall, 15 small molecules were identified (nicotine, varenicline, GTS-21, EVP-6124, ABT-089, ABT-126, MT-4666, AZD3480, PTI-125, nefiracetam, MEM3454, SSR180711, AZD1446, TC-5619 and AQW051), which were capable of modulating the activity of nicotinic cholinergic receptors. Two of the identified RCTs were still recruiting; eighteen were completed; thirteen were terminated; two were withdrawn; two had unknown status; and two were enrolling participants. A total of 16 published studies were identified reporting data on 7 of the considered molecules. 

The effects of nicotine on cognitive decline in people with dementia have been described in several scientific publications published more than 20 years ago (tab 2: Refs [35,36,37,38,40,48]). Nicotine had a positive effect on attention deficits and the neuropsychological symptoms associated with dementia, irrespective of whether the drug was administered intravenously or through a patch [36,37,40,48]. No effect of nicotine was reported on working memory, measured using cognitive tests designed to analyze the components of working memory, which are typically impaired in AD, such as attention, concentration, executive functions, verbal fluency and short- and medium-term memory. No significant improvements were also observed in other areas of higher brain functions, such as episodic or semantic memory, reasoning, spatiotemporal perception, executive functions, language, planning, learning, problem solving, which are also usually significantly impaired in people with AD [37,38]. There are currently no registered clinical trials investigating the efficacy of nicotine in patients with non-AD dementia. However, one trial reported an improvement in memory performance in people with cognitive impairment after a controlled transdermal administration of nicotine [40]. One RCT registered in 2016 (NCT02720445)—the Memory Improvement through Nicotine Dosing (MIND) study—has reached its final phase, but its research results have not yet been published. 

ABT-418, a bioisostere of nicotine, was designed to reduce nicotine’s significant side effects. It showed a positive dose-dependent effect in verbal (learning and recall) and non-verbal (spatial memory) tasks. The results are therefore in line with the positive effects of nicotine observed in working memory, which are associated with the stimulation of cholinergic receptors. However, the study did not use any standardized assessment tools to obtain a more structured profile of cognitive functions in terms of semantic or episodic memory, and no differences between groups were observed in other outcomes, such as mood, anxiety or behavioral symptoms. 

ABT-126 was originally developed by Abbott for the treatment of cognitive deficits associated with schizophrenia and AD. Our SR identified five registered RCTs and three published articles enrolling participants with mild-to-moderate AD. One study reported data from the phase II RCT NCT00948909 investigating the effect of ABT-126 (two dose groups: 5 and 25 mg/day for 12 weeks) compared to placebo and donepezil (10 mg/day) on cognitive functions [45]. No differences between ABT-126 and placebo or donepezil were observed for the primary endpoint (ADAS-Cog 11-item total score) and the secondary endpoints, including ADAS-Cog 13, MMSE, CIBIS, CIBIC-plus, NPI and ADCS-ADL. However, a dose–response analysis suggested that higher doses might be associated with a larger effect on cognitive outcomes. This was assessed in two subsequent phase II trials (NCT01527916 and NCT01676935) [46], one of which was an open-label extension study, which was prematurely terminated due to the results from the RCT. This study confirmed that higher doses of ABT-126 (25, 50, 75 mg/day)—although associated with improvement in some of the secondary outcomes (CIBIC-plus, ADCS-ADL)—were not associated with better performance compared to donepezil in the primary outcome. Another study reported efficacy data from a phase II RCT (NCT01549834) and its open-label extension (NCT01690195) [43]. Data on changes in the ADAS-Cog 11 scale scores after 24 weeks of treatment showed no significant differences between groups, irrespective of the dose (25 and 75 mg/day). Similar results were described for secondary endpoints, including cognitive (ADAS-Cog13), activities of daily living and neuropsychiatric scores. It might be worth noting that significant differences in the ADAS-Cog 11-item total score were observed between the 25 mg group and the placebo group at week 4, but not at week 8. These results did not support the efficacy of the drug as expected, leading to early termination of the open-label study. 

ABT-089—another molecule derived from nicotine and designed to reduce its side effects on peripheral systems and increase the affinity for neuronal nicotinic receptors—was originally tested for the treatment of cognitive dysfunctions in people with ADHD. Our searches identified three registered clinical trials, all phase II RCTs investigating ABT-089 in people with mild AD (tab 1; NCT00069849, NCT00555204 and NCT00809510). No published results were found for NCT00069849 or for NCT00809510, an open-label extension of NCT00555204. One published study reported data from one RCT in 2015 [42]. The study was prematurely terminated, as the primary efficacy analysis did not meet the targeted treatment effect (1.75-point improvement over placebo on the ADAS-Cog scale). No significant differences between groups were observed in any of the secondary efficacy scales (MMSE, CIBIS/CIBIC-plus, ADCS-ADL, CSDD and CDR) after 12 weeks of treatment.

The drug varenicline is approved for smoking cessation, as it reduces the acute nicotine stimulating effect on dopamine in the mesolimbic system. The potential effect of varenicline in improving cognition in mild-to-moderate AD patients was investigated in a phase II, multi-center, randomized, double-blind, placebo-controlled, proof-of-concept study (tab 1; NCT00744978) [41]. However, the results from the trial showed no differences between groups in terms of cognitive performance, as measured by the ADAS-Cog scale, and a worsening of eating habits, as assessed by the NPI, probably due to treatment-related nausea.

AZD3480 was reported to be potentially effective in improving attention and episodic memory in one clinical trial enrolling subjects with age-related subjective gradual memory impairment [81]. The results from a phase II RCT reported no improvement in the ADAS-Cog 11 score after 12 weeks of treatment, irrespective of the dose. No significant differences between groups were also observed in the secondary outcome measures (MMSE, CDR, ADCS-CGIC). Although limited improvements were observed in some subgroup analyses (see Table 2), the drug’s manufacturer announced its discontinuation due to inconsistencies in the results. 

Unlike previous drugs, PTI-125 does not directly interact with nicotine receptors but modulates their activity via filamin A [79], although concerns with respect to demonstration of the molecular mechanism have been raised [82]. The only published study reported data from the first completed open-label phase II study (NCT03748706), which—after 28 days of PTI-125 treatment—showed a significant reduction in some core markers of AD pathology (total tau, p-tau181 in CSF and plasma), neurodegeneration (neurofilament light chain, neurogranin in CSF and plasma) and neuroinflammation (YKL-40, IL-6, IL-1β and TNFα in CSF) [44]. Moreover, the results showed an increase in plasma concentrations of the soluble Aβ1-42 complex, consistent with an effect of the drug in slowing AD progression. These results led to initialization of a randomized, multiple-dose study investigating PTI-125 in patients with mild-to-moderate AD (NCT04079803). The primary endpoints included significant changes in CSF and plasma biomarkers—considered as surrogate measures of the efficacy of PTI-125 treatment in counteracting the neurodegenerative and inflammatory process—and the blood–brain barrier dysfunction associated with AD. The results were only available on CT.gov and confirmed previous findings, while no differences between groups were observed in any of the secondary outcome measures.

## 4. Discussion

Dementia is not a specific disease, but it is characterized by a broad group of symptoms defining a clinical picture—including memory loss and a decline in other mental abilities, such as thought processing, reasoning, attention, language—which are sufficiently severe to affect autonomy and self-sufficiency. Dementia is the most frequent among some central nervous system disorders, defined as primary dementias, and it is a direct result of irreversible neuronal degeneration in the brain’s complex functional circuits. Although AD is the most common form of dementia, this group of conditions also include other neurodegenerative illness, such as Lewy body dementia, PD dementia, frontotemporal dementia and prion diseases [83]. The prevalence of AD compared to other diseases had a major impact on clinical trials investigating the potential effects of nAChRs agonists. This SR identified three RCTs investigating the efficacy of nicotine—a molecule, which increases the level of dopamine in the CNS—and of the α7-selective nAChRs agonist AZD0328 in the cognitive symptoms of PD, but neither proved to be a successful neuroprotective strategy. The remaining trials enrolled participants with AD (mild to severe) or MCI, with the latter defined as a decline in cognitive and mental abilities, which, although not severely affecting the activities of daily living, is still a relevant risk factor for dementia [84,85]. Recent clinical research reports a tendency to commence treatments for AD in the prodromal stages [86], based on the assumption that neuronal damage may still be reversible at this stage. However, only three RCTs on positive modulators of ionotropic receptors were identified investigating two drugs: AQW051 and nicotine. No data were available on AQW051 either in registration databases or scientific publications, while published data were available on nicotine, showing promising results in primary and secondary outcome measures related to attention, memory and mental processing [40]. An ongoing phase II trial (NCT02720445) further investigates the effects of this drug on MCI subjects, but the results are not yet available. Overall, the results from this review suggest that studies investigating nAChRs agonists in people with MCI are still very limited, and the potential impact of these molecules on the progression from MCI or a possible phase of subjective cognitive decline to different forms of dementia still needs to be investigated. However, the results from clinical trials among participants with dementia suggest that the specific aspects of pharmacological activation of nAChRs in people with dementia raise some concerns and need to be carefully considered. A striking minority of all registered clinical trials—only 30.7% (1 phase I, 10 phase II and 1 phase III)—had the results reported in peer-reviewed publications or available on registration databases. The number of registered trials with available results for this class of drugs was significantly lower compared to other classes of drugs for AD, such as compounds involved in synaptic plasticity [87]. The availability of results, either positive or negative, allows for new hypotheses to be developed on the possible factors affecting the efficacy of drugs and for the adaptation of protocols, for example, the modification of the length of washout periods or utilization of variable doses. Therefore, leaving the results unpublished prevents from identifying the possible reasons for failure to achieve pre-defined outcomes and thus from possibly improving the protocols for new studies. As an example, ABT-126—despite not reaching the primary objective of significantly improving the ADAS-Cog 11 scores after 24 weeks of treatment compared to placebo—showed a significant effect, associated with the severity of cognitive decline, in a shorter period (4 weeks). Further studies with different observation times and different treatment durations could provide different results on the potential effect of this treatment. nAChR agonists are in fact known to induce desensitizing effects [88], which could influence their efficacy as a function of time, as reported in Florian’s study [43]. A further source of concern regarding the reliability of studies on nAChrs agonists—in particular nicotine, varenicline and PTI-125—is the limited sample size of these studies. Further studies should be carried out enrolling larger samples and adopting endpoints based on validated and widely established outcome measures, thus facilitating the generalizability and comparison of the results. Overall, the results from these trials should be interpreted, taking into account the mechanisms underlying the effects of nicotinic ligands on the nervous system. In fact, the functional state of the receptor is considered to be dependent on the concentration of the agonists, the nature of the agonists (orthosteric or allosteric) and the speed at which the exposure occurs. Moreover, it should be noted that ACh mimetics, when administered, remain in the nervous system microenvironment for a longer period of time, as they cannot be degraded as easily as ACh. Therefore, prolonged exposure to low agonist concentrations may encourage receptor desensitization, favoring the passage from a closed state to a desensitized state [89]. The desensitized conformation of the receptor generally has greater affinity for the agonists compared to the closed or opened conformation of the receptor. Another relevant aspect refers to prolonged exposure to nicotinic ligands, which may also increase the density of receptors in several animal species, including humans [90]. The phenomenon may be dependent on slow receptor recycling, as the desensitized forms of the receptor are removed very slowly from the cell membrane. When the concentration and exposure of the ligands decrease, nicotinic receptors return to the active state, resulting in hyperexcitability. Therefore, when considering experimentation with an agonist in a clinical trial, these aspects should be taken into due account to define the appropriate concentrations and timing to be used. As an example of dose-dependent effects, the RCT on AZD-3480 reported a significant effect for 20 mg at 12 weeks compared to AZD-3480 35/100 mg on some secondary cognitive outcomes (MMSE and ADCS-CGIC scores) [47]. 

When considering future clinical trials, the use of allosteric compounds could be of strategic importance in the treatment of dementia, as it could help better modulate the activity of the receptors, probably with significant advantage in terms of reduced desensitization of the receptors. It should also be mentioned that the activation of ionotropic nAChRs involves a wide range of Ca^2+^ sensitive targets, including enzymes such as cyclic-dependent AMP protein kinase (PKA) and Ca^2+^/calmodulin-dependent protein kinase. These kinases, sensitive to intracellular Ca^2+^ levels, can regulate various synaptic ion channels, as well as cytoskeletal and trafficking proteins, which control vesicle mobility and release [16]. Furthermore, calcium cell signaling mediated by nAChRs regulates gene expression in neurons, controlling the activation of transcription factors such as CREB, which plays an important role in memory and learning [17]. Therefore, it is important to consider that even a short-term treatment with nicotinic ligands can promote prolonged effects in the neurons in terms of the transcription and expression of functional proteins [31]. A positive aspect emerging from the trials included in this review is that all the considered drugs appeared to have an overall safety and tolerability profile substantially comparable to a placebo, with fewer drug-related AEs, the majority of which were considered by the investigators as mild to moderate in severity. Treatment-related SAEs were not reported, suggesting that no safety concerns were raised during treatment at the doses established in the trial protocols. However, when considering the overall profile of these drugs based on the gathered data, the positive modulators of nAChRs do not appear to be a promising option for the treatment of dementia or MCI. None of these drugs reported in Table 3 achieved the expected cognitive endpoints in mild-to-severe dementia, despite eight new trials having been registered since 2018, two of which enrolled participants with dementia associated with PD (nicotine and AZD0328: two phase II RCTs) and six investigated PTI-125 (three phase II and three phase III RCTs). The results from a small open-label phase II study among 13 participants with mild-to-moderate AD reported that this latter drug appears to modify the plasma and CSF expression profile of some protein biomarkers after 4 weeks of treatment [44]. These biomarkers are considered to be surrogate endpoints for cognitive decline, as they are associated with amyloidogenic processing and aggregation, tau hyperphosphorylation and accumulation, neuroinflammation and neurodegeneration [44]. A further open-label study enrolling 130 people with mild AD (NCT04388254) reported an improvement in ADAS-Cog 11-item scores (0.73 points). Some of the results from these RCTs have also been reported in a review by Burns and colleagues [91]. However, the full methodological aspects of the study have not yet been published, thus preventing their full quality assessment. The overall quality of the studies included in this review was medium to low, thus highlighting how future studies should attempt to be based on a more adequate and standardized methodology to evaluate the effectiveness of new and old therapeutic approaches based on the use of nAChRs agonists, including their use as adjuvants in therapies based on other agents. 

## 5. Conclusions

Dementia is a public health priority, as, according to the World Health Organization, more than 55 million people will be affected by this condition in 2023, making it the seventh leading cause of death. The social and economic costs of this so-called silent pandemic are enormous and support the urgent need to find effective therapeutic treatments. Even though dementia is a heterogeneous condition from a nosological point of view, the higher frequency of cases attributable to AD (60/70%) has historically directed many pharmacological studies toward the treatment of this condition, as is the case with the trials analyzed in this SR, having nAChRs as their therapeutic target. These channels have many characteristics, both in terms of their localization in brain areas and in terms of their role in functional processes, which make them suitable to be modulated for therapeutic purposes in case of neurodegeneration. However, in contrast to this theoretical predisposition, the use of nAChRs agonists has led to the termination of many clinical phase II/III trials. The results from most of these trials have not been published, thus leaving uncertainties on both the safety and efficacy of these drugs. Based on the published results, no concerns appear to have been raised on their tolerability, except for some sporadically occurring gastrointestinal disorders. The data reported in this SR seem to confirm that the lack of success in clinical efficacy is the main reason, which led to discontinuation of research on this class of drugs. It should be noted, however, that although, in many cases, the primary endpoints were not met, some secondary results appear to be encouraging, suggesting that a possible role of nAChRs agonists in treating symptoms of dementia might still be considered. On this basis, future trials should be designed considering as endpoints those secondary outcome measures, which showed promising results in slowing the neurodegenerative mechanisms. The relationship between this class of drugs and the progression of dementia from cognitive decline to dementia has not yet been fully investigated, with published data being available from only one clinical trial among subjects with mild cognitive impairment treated with nicotine and reporting interesting results, which still need to be confirmed. Finally, the chemical–pharmacological properties, functional effects, such as receptor desensitization, as well as dosing protocols need to be further investigated in high-quality trials before evidence-based conclusions can be drawn on the efficacy and safety of nAChR agonist candidates in dementia.

## Figures and Tables

**Table 1:** Identifiers, interventions, main features and outcomes of selected trial protocols from Clinical Trials.gov and European Clinical Trials Register.

Identifier	Intervention	Duration	Placebo	Estimated Enrollment (Participants)	Age (Years)	Diagnosis	MMSE at Baseline	Primary Outcome	Secondary Outcome	Status
1 NCT02720445 Phase 2	Nicotine Transdermal Patch	FP: 2016 LUP: 2023	Y	380	55–90	MCI	24–30	Conners’ Continuous Performance Task (CPT)	MCI-CGIC, CBB, NYU Paragraph Recall, CDR-SOB, GDS, ADCS-ADL, OASR/OABCL, CSF Biomarkers, vMRI	Recruiting, No Study Results
2 NCT00091468 Phase 1	Nicotine Transdermal Patch	FP: 2004 LUP: 2008	Y	75	55–90	MCI	24–30	Safety	Cognitive performance, global function	Unknown, No Study Results
3 NCT03865121 Phase2	Nicotine Transnasal	FP: 2019 LUP: 2019	N	6	>60	PD	/	MDS-UPDRS	Efficacy, MDS-UPDRS	Completed, No Study Results
4 NCT01560754 Phase 2	Nicotine Transdermal	FP: 2012 LUP: 2015	Y	160	>30	Early PD	/	UPDRS I-III	UPDRS I-III, (PDQ-8), AEs, SCOPA-COG, BDI-II, PDSS	Unknown, Results Submitted
5 NCT00744978 Phase 2	Varenicline	FP: 2008 LUP: 2011	Y	66	55–85	Mild-to-moderate AD	14–26	ADAS-Cog 75	ADAS-Cog 75, ADAS-Cog 70, CGI-I, NPI, CogState, CPAL,	Completed, With Results
6 NCT00414622 Phase 2	GTS-21 (DMXB-A)	FP: 2006 LUP: 2007	Y	60	50–80	AD	/	CDR	ADAS-Cog	Completed, Results Submitted
7 NCT02246075 Phase 2	EVP-6124/memantine encenicline	FP: 2014 LUP: 2015	Y	0	55–85	AD, dementia cognition	12–26	Safety, tolerability	MMSE	Withdrawn (Forum has decided not to proceed with this study at this time.)
8 NCT00766363 Phase 2	EVP-6124	FP: 2011 LUP: 2012	Y	49	50–90	Mild-to-moderate AD	18–26	Safety and tolerability	PK	Completed, With Results
9 NCT01073228 Phase 2	EVP-6124	FP: 2010 LUP: 2014	Y	409	50–85	Mild-to-moderate AD	14–24	ADAS-cog-13 Time Frame: Day -7	ADAS-cog-13 Time Frame: Day—4, Controlled Oral Word Association TEìest, Clinical Dementia Rating Scale Sum of Boxes, Alzheimer’s Disease Cooperative Study-Activities of Daily Living, NPI, MMSE	Completed, No Study Results
10 NCT02004392 Phase 3	EVP-6124	FP: 2013 LUP: 2016	Y	348	55–85	AD, dementia	/	Safety	MMSE, NPI, EQ-5D, RUD-Lite 3.3, ZBI	Terminated (Study has been suspended due to clinical hold.)
11 NCT01969123 Phase 3 Eudra: 2013-002618-10	EVP-6124	FP: 2013 LUP: 2016	Y	474	55–85	AD, dementia	14–24	ADAS-Cog-13, CDR-SB, safety, tolerability	DAD, NPI, MMSE, COWAT	Terminated (Study has been suspended due to clinical hold.)
12 NCT01969136 Phase 3 Eudra: 2013-002653-30	EVP-6124	FP: 2013 LUP: 2016	Y	403	55–85	AD, dementia	14–24	ADAS-Cog-13, CDR-SB, safety, tolerability	DAD, NPI, MMSE, COWAT	Terminated (Study has been suspended due to clinical hold.)
13 NCT00809510 Phase 2 Non-Randomized Open-Label	ABT-089 Pozanicline	FP: 2008 LUP: 2011	N	63	55–90	AD	/	Safety	ADAS-Cog, MMSE, QoL-AD, CIBIC-Plus	Terminated
14 NCT00555204 Phase 2	ABT-089	FP: 2007 LUP: 2011	Y	337	55–90	AD	12–26	Safety and tolerability	PK, PD	Terminated
15 NCT00069849 Phase 2	ABT-089	FP: 2003 LUP: 2006	N	64	50–85	AD	12–26	MMSE	/	Terminated
16 NCT01676935 Phase 2 Open-label Eudra: 2011-004780-75	ABT-126 Nelonicline	FP: 2012 LUP: 2021	N	349	55–90	AD	/	AE, laboratory data, vital signs, physical examinations, brief neurological and psychiatric assessments, Columbia-Suicide Severity Rating Scale, Cornell Scale for Depression in Dementia, electrocardiogram	/	Terminated (Data obtained from the M11-427 study are not critical to the continued evaluation of ABT-126.)
17 NCT01690195 Phase 2 Open-label Eudra: 2012-000537-39	ABT-126	FP: 2012 LUP: 2021	N	343	55–90	AD	/	AE, laboratory data, vital signs, physical examinations, brief neurological and psychiatric assessments, Columbia-Suicide Severity Rating Scale, Cornell Scale for Depression in Dementia, electrocardiogram	/	Terminated
18 NCT01549834 Phase 2 Eudra: 2011-004849-40	ABT-126	FP: 2012 LUP: 2014	Y	434	55–90	AD	12–24	MMSE, ADCS-ADL, DEMQOL, CIBIC-plus, NPI, PPQSA, RUD-Lite, EuroQol-5D Questionnaires WMS-III, working memory index	/	Completed
19 NCT01527916 Phase 2 Eudra: 2011-002004-32	ABT-126	FP: 2012 LUP: 2014	Y	438	55–90	AD	10–24	ADAS-Cog	ADCS-ADL, DEMQOL, CIBIC-plus, NPI, MMSE, PPQSA, WMS-III, EuroQol-5D Questionnaires	Completed
20 NCT00948909 Phase 2 Eudra: 2009-011424-64	ABT-126/Donepezil	FP: 2009 LUP: 2013	Y	274	55–90	AD	10–24	ADAS-Cog	ADCS-ADL, MMSE, QoL-AD, CIBIC-plus, NPI, CSDD, ADAS-Cog	Completed
21 NCT01764243 Phase 2	MT-4666	FP: 2013 LUP: 2015	Y	450	50–85	Probable AD	10–24	ADAS-cog-13	CDR-SB, ADCS-ADL, ADAS-cog-11, MMSE, NPI, Modified Crichton Scale	Completed, No Results
22 NCT02327182 Phase 3	MT-4666	FP: 2014 LUP: 2015	N	117	55–85	AD	14–24	Safety	MMSE, NPI	Terminated (This study was terminated due to the benefit–risk balance of MT-4666.)
23 NCT01466088 Phase 2 Eudra: 2011-000487-10	AZD3480/Donepezil	FP: 2011 LUP: 2015	N	386	60–85	AD	12–22	ADAS-Cog, CIBIC+, ADCS-ADL	NPI, MMSE, ADRQL	Completed, No Study Results
24 NCT00501111 Phase 2 Eudra: 2007-000835-24	AZD3480/Donepezil	FP: 2007 LUP: 2014	Y	659	60–85	AD	/	ADAS-Cog	CDR, MMSE, ADCS-CGIC	Completed, Results Submitted
25 NCT04079803 Phase 2	PTI-125	FP: 2020 LUP: 2023	Y	64	50–85	Mild-to-moderate AD	16–26	Change from baseline in CSF Abeta42, Total Tau, P-tau181, Neurogranin, Neurofilament Light Chain, YKL-40	Cognitive test assessing episodic memory, Cognitive assessment of spatial working memory, CSF IL-6, sTREM2, HMGB1, Albumin, IgG	Completed, With Results
26 NCT04388254 Phase 2	PTI-125	FP: 2020 LUP: 2023	N	200	50–85	Mild-to-moderate AD	16–26	Change from baseline in CSF P-tau, Total Tau, Abeta42, Neurofilament Light Chain, Neurogranin, YKL-40, Soluble TREM2 and HMGB1	NPI, Change from baseline in Total Tau, P-tau, Abeta42, P-tau181, Neurofilament Light Chain, Neurogranin, YKL-40, Soluble TREM2 and HMGB1 during open-label period, Cerebrospinal fluid biomarkers of AD	Active, Not Recruiting, No Study Results
27 NCT05026177 Phase 3	PTI-125 REFOCUS-ALZ	FP: 2021 LUP: 2023	Y	1083	50–87	AD	16–27	ADAS-Cog12, ADCS-ADL	iADRS, NPI, MMSE, CDR-SB, ZBI, CSF Neurogranin, Neurofilament Light Chain, Total Tau, P-tau181, Soluble triggering receptor expressed on myeloid cells 2 (sTREM2), and Aβ1-42, MRI, amyloid and tau PET, Plasma biomarkers P-tau181 and Neurofilament Light Chain, Plasma biomarker SavaDx	Recruiting, No Study Results
28 NCT05575076 Phase 3 Open-label	PTI-125	FP: 2022 LUP: 2023	N	1600	51–89	AD	/	AE	/	Enrollment By Invitation, No Study Results
29 NCT04994483 Phase 3	PTI-125 RETHINK-ALZ	FP: 2021	Y	750	50–87	Mild-to-moderate AD	16–27	ADAS-Cog 22 items	Change from baseline in iADRS, NPI, MMSE, CDR-SB, ZBI, Plasma phospho-tau181 and/or phospho-tau217, Neurofilament Light Chain, Plasma SavaDx biomarker (to detect altered filamin A)	Active, Not Recruiting, No Study Results
30 NCT03748706 Phase 2 Open-label	PTI-125 Simufilan	FP: 2018 LUP: 2021	N	13	50–85	AD	16–24	Cmax, Tmax, Clast, Tlast, AUClast, Plasma half-life	CSF Biomarkers, SavaDx Biomarker	Completed, With Results
31 NCT00001933 Phase 2	Nefiracetam	FP: 1999 LUP: 2008	Y	50	Child, Adult, Older Adult	AD	12–25	Efficacy, safety, tolerability	Nefiracetam enhances the activity of nicotinic acetylcholine receptors by interacting with a protein kinase C pathway and accelerates acetylcholine turnover and release. Efficacy in patients with mild-to-moderate dementia will be assessed through application of standardized neuropsychological test instruments	Completed, No Study Results
32 NCT00884507 Phase 2 Eudra: 2008-004012-13	RO5313534/Donepezil MEM3454	FP: 2009 LUP: 2016	Y	389	>50	AD	13–22	ADAS-Cog	CANTAB tests, MMSE total score, ADCS CGIC, Behave-AD-FW, ADCS-ADL, Zarit Burden interview, AEs, lab parameters, suicidal risk, concomitant medications, physical and neurological examinations	Completed, No Study Results
33 NCT00454870 Phase 2	RO5313534	FP: 2007 LUP: 2008	Y	80	50–80	AD	16–26	Safety, efficacy, PK, tolerability	/	Completed, No Study Results
34 NCT00602680 Phase 2 EUDRA: 2007-001639-80	SSR180711C/Donepezil	FP: 2008 LUP: 2009	Y	1	55–90	AD	/	Change in cognitive performance	Cognitive, global, function and behavioral assessments	Terminated (Insufficient expected benefit–risk balance), No Study Results
35 NCT01039701 Phase 2 EUDRA: 2009-015525-37	AZD1446/Donepezil	FP: 2009 LUP: 2010	Y	99	60–85	Mild-to-moderate AD	/	AEs	PK of AZD1446 as an add-on treatment to donepezil in AD patients, effects of three-dose regimens of AZD1446 compared to placebo as an add-on treatment to donepezil on changes in global functioning using ADCS-CGIC	Completed, Results Submitted
36 NCT01125683 Phase 2 EUDRA: 2010-018273-38	AZD1446/Donepezil	FP: 2010 LUP: 2011	Y	40	55–85	AD	18–24	Effect of single and multiple dosing of AZD1446 and a single dose of donepezil on quantified qEEG and ERP in patients with mild-to-moderate AD	Measure the relationship between plasma concentration of AZD1446/donepezil and qEEG and ERP, evaluate the correlation between changes in qEEG/ERP and changes in Cognition, Safety, AEs, Vital Signs, ECG, Clinical Chemistry, Hematology, Urinalysis and Physical Examination	Terminated (Poor recruitment), Results Submitted
37 NCT01254448 Phase 1	TC-5619	FP: 2010 LUP: 2013	Y	38	55–80	AD	12–22	AEs, safety and tolerability	PK, Markers of inflammation in cerebrospinal fluid and plasma	Completed, No Study Results
38 NCT00582855 Phase 2 EUDRA: 2007-001846-42	AQW051	FP: 2007 LUP: 2016	Y	54	55–85	Mild AD, aMCI	/	Validated computerized cognitive assessment scores	ADAS-Cog, Quality of Life-Alzheimer Disease Scale and the Disability Assessment for Dementia Scale	Terminated, No Study Results
39 NCT04810104 Phase 2 EUDRA: 2019-002423-15	AZD0328	FP: 2021 LUP: 2022	Y	0	50–80	PD with MCI	/	Attentional Intensity Index	Attentional Intensity Index, Sustained Attention Index, Working Memory Index, Episodic Memory Index, Memory Speed Retrieval Index, MoCA, MDS-UPDRS Part III, Non-Motor Symptom Scale, MCI-CGIC, HADS, AEs, SAEs, ECG	Withdrawn Due To COVID-19

Abbreviations: MCI: Mild Cognitive Impairment; AD: Alzheimer’s Disease; CSF: Cerebrospinal Fluid; CPT: Conners’ Continuous Performance Task; MCI-CGIC: Mild Cognitive Impairment—Clinical Global Impression of Change; CBB: Cogstate Brief Battery; NYU: New York University; CDR: Clinical Dementia Rating Scale; SOB (CDR-SB/CDR-SOB): Sum of Boxes; GDS: Geriatric Depression Scale; ADCS-ADL: Alzheimer’s Disease Cooperative Study—Activities of Daily Living Inventory; OASR: Older Adult Self Report; OABCL: Older Adult Behavior Checklist; CSF: Cerebral Spinal Fluid; vMRI: Volumetric Magnetic Resonance Imaging; ADAS-Cog 75: Alzheimer’s Disease Assessment Scale—Cognitive Subscale 75; ADAS-Cog 70: Alzheimer’s Disease Assessment Scale—Cognitive Subscale 70; CGI-I: Clinical Global Impression—Improvement; NPI: Neuropsychiatric Inventory; CogState: Computerized Test Battery for Cognition; CPAL: Continuous Paired Associate Learning; MMSE: Mini-Mental State Examination; Qol-AD: Quality of Life in Alzheimer’s Disease; CIBIC-Plus: Clinician’s Interview-Based Impression of Change Plus; AE: Adverse Event; DEMQOL: Dementia Quality of Life; PPQSA: Partner-Patient Questionnaire for Shared Activities; RUD-Lite: Resource Use in Dementia; WMS-III: Wechsler Memory Scale-III; CSDD: The Cornell Scale for Depression in Dementia; EQ-5D: Quality of Life Using the EuroQol-5D; ZBI: Zarit Burden Interview; ADAS-Cog-13: the 13-item Alzheimer’s Disease Assessment Scale-Cognitive subscale; DAD: Disability Assessment for Dementia; COWAT: Controlled Oral Word Association Test; C-max: Maximum Concentration; Tmax: Time to Maximum Concentration; AUC: Area Under the Curve; ADAS-Cog-11: the 11-item Alzheimer’s Disease Assessment Scale-Cognitive subscale; ADRQL: Alzheimer’s Disease Related Quality of Life; ADCS-CGIC: Alzheimer’s Disease Cooperative Study—Clinical Global Impression of Change; SIB: Severe Impairment Battery; ADCS-ADLsev: Alzheimer’s Disease Cooperative Study—Activities of Daily Living (Severe); PK: Population Pharmacokinetic; iADRS: Integrated Alzheimer’s Disease Rating Scale; Clast: Last Quantifiable Plasma Concentration; Tlast: Time to Last Quantifiable Plasma Concentration; AUClast: Area Under the Curve.

**Table 2:** Summary of the main characteristics and results of the RCT with available data.

Reference	Study Design	Study Population	Diagnostic Criteria	Objective(s)	Treatment Duration	Intervention	AEs/SAEs	Number of Dropouts	Efficacy Results
ND									
Newhouse et al., 1990 [35]	A single-blind investigation	11 nonsmoking AD patients (7 F, 4 M; age 58–80; mean age 65.9 + 8.3)	DSM-III-R criteria (American Psychiatric Association, 1987), NINCDS-ADRDA, Global Deterioration Scale	Neurohormonal and behavioral responses to intravenous (IV) nicotine	60 min	Nicotine 0.125, 0.25 and 0.5 I.tg/kg/min and placebo (the equivalent volume of 0.9% saline)	/	/	Anxiety self-ratings vs. placebo (*p* = 0.004) after the 0.5 μg dose at +30 min (*p* < 0.01) and +60 rain (*p* < 0.05) after 0.25 μg (*p* < 0.05) +60 min intragroup (*p* = 0.008) 0.5 and 0.25 μg doses. NIMH Self-Rating Scale (*p* < 0.05). VAS scale, the anxiety (*p* < 0.05) intergroup vs. placebo 0.25 μg (+30 min) 0.5 μg (+30 and 60 min)
Snaedal et al., 1996 [36]	Placebo-controlled, double-blind study with a cross-over design	24 subjects with probable AD (mean age 80.4 ± 6.2)	NINCDS-ADRDA, MMSE 12–28	Effects of nicotine transdermal patch on cognitive functions	4 weeks + 2-week washout	Nicotine dermal plasters 21 mg	2 AEs in intervention group	3 dropouts	RAVLT intergroup vs. placebo: *p* < 0.05, short-term memory placebo vs. intervention *p* < 0.01
Sahakian et al., 1994 [37]	Single-blind, placebo-controlled study	22 subjects with probable AD, 24 normal elderly subjects	NINCDS-ADRDA for AD, CDRS	Effects of three acute doses of nicotine (0.4, 0.6, 0.8 mg) administered to participants with AD and normal controls	40 min	(1) Subcutaneous nicotine 0.4 mg; (2) Subcutaneous nicotine 0.6 mg; (3) Subcutaneous nicotine 0.8 mg; (4) Placebo.	/	/	RVIP intergroup treatment vs. placebo: *p* < 0.001, intergroup 0.4 mg vs. placebo *p* > 0.05, DRMLO intergroup intervention vs. placebo *p* < 0.02
White et al., 1998 [38]	Placebo-controlled, double-blind cross-over study	8 subjects with mild-to-moderate AD	NINCDS-ADRDA, CDR	Effects of nicotine patch on cognitive performance	4 weeks + 2-week washout	(1) Nicotine patch 5 mg; (2) Nicotine patch 10 mg; (3) Placebo.	/	2 dropouts	ADAS-cognitive placebo: 23 ± 4.3 nicotine 22.5 ± 3.8 *p* = 0.65, ADAS-non-cognitive placebo 4.8 ± 1.7 nicotine 4.8 ± 1.2 *p* = 0.92, PDS placebo 108.5 ± 16.9, nicotine 106.4 ± 12.0 *p* = 0.71, ADLs placebo 9.8 ± 1.6 nicotine 9.9 ± 1.5 *p* = 0.95, IADLs placebo 22.6 ± 2.2 nicotine 22.2 ± 1.9 *p* = 0.56, CGI-caregiver placebo 4.0 ± 0.3 nicotine 3.8 ± 0.2 *p* = 0.1, CGI-physician placebo 4.0 ± 0.2 nicotine placebo 3.9 ± 0.1 *p* = 0.38, Simple reaction time placebo 923 ± 164 nicotine 748 ± 86 *p* = 0.17, Choice reaction time placebo 997 ± 169 nicotine 1024 ± 178 *p* = 0.82, Spatial processing placebo 4543 ± 675 nicotine 4746 ± 438 *p* = 0.60, Delayed matching placebo 51.5 ± 8.2 nicotine 49.5 ± 4.8 *p* = 0.69, Stroop color and word test placebo −1.1 ± 1.4 nicotine 0.5 ± 1.4 *p* = 0.22, Sternberg memory test placebo 54.7 ± 2.9 nicotine 58.5 ± 4.9 *p* = 0.18, Digit span placebo 11.4 ± 1.6 nicotine 10.7 ± 1.6 *p* = 0.14. ADAS-cognitive intergroup −0.5 (0.5), ADAS-non-cognitive 0 (0.5), PDS placebo −2.1 (4.9), ADLs 0.1 (0.1) 9.8, IADLs −0.4 (0.3), CGI-caregiver −0.2 (0.1), CGI-physician placebo −0.1 (0.1), Simple reaction time placebo −175 (11), Choice reaction time placebo 27 (11), Spatial processing placebo 203 (237), Delayed matching placebo −2 (4.6), Stroop color and word test 1.6, Sternberg memory test placebo 4.2 (2.0), Digit span placebo −0.7. Composite attention nicotine administration *p* < 0.025. The subjects’ scores rose from 1.19 ± 0.47 (mean ± SEM) with placebo to 1.55 ± 0.47 with nicotine. Nicotine-induced decline in the response bias measure *p* < 0.07.
Potter et al., 1999 [39]	Double-blind, within-subjects, repeated-measures design	6 subjects with probable AD (Gender: N.D.; Mean age: 72.7 ± 10.7)	NINDS-ADRDA criteria MMSE: 21.4 ± 3.0 Mean global deterioration: 3.2	SRT, RAT, SMT, CPT, Psychomotor ability, SAV	4 days	(1) Placebo; (2) ABT-418 6 mg; (3) ABT-418 12 mg; (4) ABT-418 23 mg. Four dosing days, each separated by 48 h. Drug or placebo administered continuously for 6 h	Not reported	None	SRT: Placebo = −3.8 ABT-418 6 mg = −1.0 ABT-418 12 mg = +0.5 ABT-418 23 mg = +2.8 *p* < 0.05 vs. placebo SRT recall failure: Placebo = +4.5 ABT-418 6 mg = −2.0 ABT-418 12 mg = −0.3 ABT-418 23 mg = −2.7 SMT: Placebo = −5.67 ABT-418 6 mg = −4.17 ABT-418 12 mg = +0.33 ABT-418 23 mg = +1.33 RAT: Placebo = +2.34 ABT-418 6 mg = −4.50 ABT-418 12 mg = +1.23 ABT-418 23 mg = −2.33 CPT: Placebo = −0.018 ABT-418 6 mg = −0.027 ABT-418 12 mg = −0.084 ABT-418 23 mg = −0.075 SAV anxiety: Placebo = +2.5 ABT-418 6 mg = −13.67 ABT-418 12 mg = −3.17 ABT-418 23 mg = −6.67 SAV fear: Placebo = +8.5 ABT-4186 mg = +9.5 ABT-418 12 mg = +9.34 ABT-418 23 mg = −4.67
**Phase I**									
Newhouse et al., 2012 [40] (NCT00091468)	Double-blind, parallel-group, placebo-controlled, randomized, pilot clinical trial	100 subjects with MCI recruited, 74 randomized: 45 M, 29 F. Treatment: 25 M, 14 F. Mean age: 76.2 ± 8.5. Placebo: 20 M, 15 F. Age: 75.7 ± 6.5	Logical Memory II Subscale (Delayed Paragraph Recall) from the Wechsler Memory Scale–Revised, MMSE 24–30, CDR 0.5–1.0	Preliminary safety and efficacy of transdermal nicotine in cognitive performance and clinical outcomes in participants with MCI	6 months	(1) Transdermal nicotine patch 5 mg, 10 mg, 15 mg; (2) Placebo.	Total AEs: (1) Treatment: 82. (2) Placebo: 52.	Treatment: 5. Placebo: 2.	Continuous performance: No. of omissions in intragroup treatment: −0.5 (1) Intragroup Placebo: 13.3 (8.9) Intergroup Treatment vs. Placebo: −2.57 (1.35) Percent of omissions: Intragroup treatment: 0.2 (0.3) Intragroup Placebo: 4.1 (2.7) Intergroup treatment vs. Placebo: −0.8 (0.4) Number of commissions intragroup treatment: −1.4 (0.1) Intragroup Placebo: −1.5 (0.3) Intergroup Treatment vs. Placebo: −0.4 (0.6) Percent of commissions intragroup treatment: −4 (0.2) Intragroup Placebo: −23.7 (1.6) Intergroup Treatment vs. Placebo: 15.5 (1.2) Hit reaction time intragroup treatment: −33 (15) Intragroup Placebo: 7 Intergroup Treatment vs. Placebo: −21 (2) Paragraph recall immediate intragroup treatment: −1.5 Intragroup Placebo: −0.5 Intergroup Treatment vs. Placebo: −0.6 Paragraph recall delayed intragroup treatment: −0.2 Intragroup Placebo: −0.3 Intergroup Treatment vs. Placebo: 0 Cognitive Drug Research Battery individual item scores simple reaction time intragroup treatment: 20 (0.2) Intragroup Placebo: −5 (3) Intergroup Treatment vs. Placebo: −3 (4) Cognitive Drug Research Battery individual item scores choice reaction time intragroup treatment: −9 (0.2) Intragroup Placebo: −10 (3) Intergroup Treatment vs. Placebo: −23 (8) Delayed picture recognition sensitivity intragroup treatment: −0.05 (0.01) Intragroup Placebo: −0.03 (0.01) Intergroup Treatment vs. Placebo: 0.06 (0.01) Delayed word recognition sensitivity intragroup treatment: 0.04 (0.02) Intragroup Placebo: −0.03 Intergroup Treatment vs. Placebo: 0.01 (0.01) Spatial memory reaction time intragroup treatment: −40 (10) Intragroup Placebo: −82 (26) Intergroup Treatment vs. Placebo: −139 (88) Spatial memory sensitivity intragroup treatment: 0 Intragroup Placebo: −0.02 Intergroup Treatment vs. Placebo: 0.09 (0.02) Digital vigilance accuracy intragroup treatment: −1.69 (0.7) Intragroup Placebo: −1.9 (0.3) Intergroup Treatment vs. Placebo: −1.09 (0.3) Digital Vigilance reaction time intragroup treatment: 9 (2) Intragroup Placebo: −14 Intergroup Treatment vs. Placebo: −6 (3) Immediate word recall intragroup treatment: 0.02 (0.07) Intragroup Placebo: −0.02 (0.04) Intergroup Treatment vs. Placebo: −0.19 (0.04) Delayed word recall intragroup treatment: 0.66 (0.09) Intragroup Placebo: 0.24 (0.01) Intergroup Treatment vs. Placebo: 0.10 (0.08)
**Phase II**									
Kim et al., 2014 [41] (NCT00744978)	Multi-center, double-blind, two-period, cross-over, randomized study	66 subjects with probable AD: 32 varenicline to placebo, age 71.5 (55–85); 34 placebo to varenicline, age 73.9 (61–85)	NINCDS-ADRDA, MMSE 14–26	Effect of varenicline on cognition in participants with mild-to-moderate probable AD	12 weeks + 3-week washout	(1) Placebo; (2) Varenicline 1 mg.	Period 1: Treatment AEs 4 (12.9%) Period 2: Treatment AEs 1 (3.2%) Period 2: Placebo group: AEs 1 (3.2%)	Treatment group Lost to follow-up 1 (3.2%) Placebo group: Refusal to participate 2 (6.1%) Other 2 (6.1%)	ADAS-Cog 75 intergroup treatment vs. placebo: –0.42, *p* = 0.3873 ADAS-Cog 70 intergroup treatment vs. placebo: –0.37 *p* = 0.4339 NPI intergroup treatment vs. placebo: 1.28 *p* = 0.0468 NPI caregiver distress intergroup treatment vs. placebo: 0.42 *p* = 0.2624 CGI-I intergroup treatment vs. placebo: 0.00 *p* = 0.9745 CogState tasks intergroup treatment vs. placebo: Visual learning –0.01 *p* = 0.5008, Detection −0.00 *p* = 0.7226, Identification 0.00 *p* = 0.8525, One-back working memory –0.02 *p* = 0.5450, Continuous paired associate learning, n errors –1.31 *p* = 0.6251 ADAS-Cog 75 intragroup treatment: −1.14 placebo: –0.73 ADAS-Cog 70 intragroup treatment: −1.05 placebo: –0.61 NPI intragroup treatment: 1.01 placebo: −0.44 NPI caregiver distress scores intragroup treatment: 0.00 placebo: −0.31
Lenz et al., 2015 [42] (NCT00555204)	Phase 2, placebo-controlled, double-blind, multi-center study	337 subjects with probable AD or amnestic MCI: (1) Placebo: 101 (60 F/41 M, age 75.0 ± 8.56); (2) ABT-089 5 mg: 12 (7 F/5 M, mean age 71.3 ± 9.85); (3) ABT-089 10 mg: 19 (12 F/7 M, mean age 76.4 ± 6.24); (4) ABT-089 15 mg: 34 (18 F/ 16 M, mean age 77.8 ± 7.48); (5) ABT-089 20 mg: 34 (20 F/14 M, mean age 75.6 ± 7.56); (6) ABT-089 30 mg: 57 (28 F/30 M, mean age 75.4 ± 7.55); (7) ABT-089 35 mg: 77 (39 F/38 M, mean age 76.0 ± 7.87).	NINDS-ADRDA criteria, MMSE: 12–26	Safety and efficacy of ABT-089 in cognitive function MMSE, ADAS-cog, CIBIC-plus, ADCS-ADL, NPI, CSDD, CDR	12 weeks	(1) Placebo; (2) ABT-089 5 mg; (3) ABT-089 10 mg; (4) ABT-089 15 mg; (5) ABT-089 20 mg; (6) ABT-089 30 mg; (7) ABT-089 35 mg.	AEs: (1) 61 (2) 138 SAEs: Not reported	106	ADAS-Cog: ABT-089 5 mg = −1.87 ABT-089 10 mg = +0.22 ABT-089 15 mg = +0.43 ABT-089 20 mg = −0.23 ABT-089 30 mg = +0.03 ABT-089 35 mg = +0.10 MMSE: ABT-089 5 mg = −0.14 ABT-089 10 mg = −0.89 ABT-089 15 mg = −0.59 ABT-089 20 mg = −0.43 ABT-089 30 mg = −0.19 ABT-089 35 mg = −0.12 CSDD: ABT-089 5 mg = +0.29 ABT-089 10 mg = +0.44 ABT-089 15 mg = +0.08 ABT-089 20 mg = −0.26 ABT-089 30 mg = +0.56 ABT-089 35 mg = +0.28 ADCS-ADL: ABT-089 5 mg = −0.21 ABT-089 10 mg = −0.03 ABT-089 15 mg = +2.26 ABT-089 20 mg = +0.68 ABT-089 30 mg = −0.10 ABT-089 35 mg = −0.72 NPI: ABT-089 5 mg = +3.77 ABT-089 10 mg = +1.80 ABT-089 15 mg = −2.15 ABT-089 20 mg = −1.15 ABT-089 30 mg = +0.99 ABT-089 35 mg = +1.50 CIBIC-Plus: ABT-089 5 mg = +0.09 ABT-089 10 mg = −0.09 ABT-089 15 mg = +0.01 ABT-089 20 mg = −0.08 ABT-089 30 mg = −0.01 ABT-089 35 mg = −0.02
Florian et al., 2016 [43] (NCT01549834)	Phase 2, randomized, double-blind, placebo-controlled multi-center study	565 subjects: 434 (76.8%) mild-to-moderate Alzheimer’s Disease, Age 55–90 (1) ABT-126 25 mg:143 (2) ABT-126 75 mg:145 (2) Placebo:146	NINCDS/ADRDA criteria, MMSE score 12–24	Efficacy of ABT-126 as add-on therapy to AChEIs	24 weeks	(1) ABT-126 25 mg (2) ABT-126 75 mg (3) Placebo once daily	At least one AE was reported for 294 subjects (67.7%) in the study ABT-126 25 mg group, 7 subjects (4.9%), ABT-126 75 mg group, 11 subjects (7.6%), placebo group 9 subjects (6.2%). ABT-126 75 mg group experienced psychiatric disorders compared with the placebo group 3.4% vs. 0% *p* = 0.030, two-sided	57 (13.1%) prematurely discontinued study drug	ADAS-Cog 11-item total score Adjusted mean 1.37 (placebo) 0.57 (25 mg ABT-126, *p* = 0.087, one-sided). ADAS-Cog 11-item total score for the 25 mg ABT-126 group was significant compared with placebo at week 4 (−0.54 vs. 0.66 *p* = 0.010, one-sided). The adjusted mean for ADAS-Cog 11-item total score in the mild AD population (MMSE ≤ 19) subgroup was significantly improved for 25 mg ABT-126 (*p* < 0.05, one-sided) compared with placebo at weeks 4 (−0.95 vs. 0.68), 8 (1.08 vs. 0.41), 12 (−0.96 vs. 0.41) and 24 (0.62 vs. 0.81)
Wang et al., 2020 [44]	Blinded, randomized, placebo-controlled, clinical trial, open-label, phase 2a, safety, pharmacokinetics and biomarker study	13 mild-to-moderate Alzheimer’s Disease patients, age 50–85 (9 F, 4 M; 3 black; 10 white; 6 Hispanic; 7 non-Hispanic)	Mini-Mental State Exam ≥ 16 and ≤24 with a cerebrospinal fluid total tau/Aβ1-42 ratio ≥ 0.30	Safety, tolerability and effect of PTI-125 on participants with mild-to-moderate AD	28 days	(1) 100 mg oral PTI-125 b.i.d. for 28 consecutive days; (2) Placebo.	None	None	Total tau, neurogranin and neurofilament light chain decreased by 20%, 32% and 22%, respectively. P-tau (pT181) decreased 34%. Cerebrospinal fluid biomarkers YKL-40 and interleukin-6, interleukin-1ß and TNFα decreased 9%, 14%, 11% and 5%, respectively. Plasma All reductions were of slightly lower magnitude in plasma, except for neurogranin, which was reduced 40.7%. Tau phosphorylation at pT181-tau, pS202-tau and pT231-tau was significantly reduced in plasma by 12.5%, 14.0% and 16.3%, respectively.
Gault et al., 2015 [45] (NCT00948909) Phase II	Phase 2, double-blind, parallel, randomized, placebo- and active-controlled study	274 subjects with probable AD, age 73.9 ± 7.92 (1) placebo: 68 age: 73.6 ± 8.23 (2) ABT-126 5 mg: 68 age 74 ± 7.47 (3) ABT-126 25 mg: 75.7 ± 7.35 (4) Donepezil 10 mg: 68 age 72.4 ± 8.42	Age 55–90 years National Institute of Neurological and Communicative Disorders and Stroke and the Alzheimer’s Disease and Related Disorders Association criteria for probable AD, MMSE score 10–24, CSDD 10 Modified Hachinski Ischemic Scale score of 4	11-item ADAS-Cog, 13-item ADAS-Cog, MMSE, CIBIS, Neuropsychiatric Inventory, ADSC-ADL, Safety	12 weeks	(1) Placebo (2) ABT-126 5 mg (3) ABT-126 25 mg (4) Donepezil 10 mg	(1) AEs: 2 (2) AEs:1 (3) AEs:1 (4) AEs:5	(1) 1 (3) 3 (4) 1	11-item ADAS-Cog total score vs. placebo: ABT-126 25 mg: −1.86 ± 0.64 (*p* = 0.95) 11-item ADAS-Cog total score maximum likelihood repeated-measures vs. placebo: ABT-126 25 mg: −0.84 at week 4 (90% confidence interval [CI] −1.92 to 0.23, *p* = 0.098), −1.11 at week 8 (90% CI −2.47 to 0.26, *p* = 0.091) and −1.14 at week 12 (90% CI −2.65 to 0.37, *p*= 0.107) 13-item ADAS-Cog total score vs. placebo: ABT-126 25 mg: −2.60 ± 0.75 (*p* = 0.042) 13-item ADAS-Cog total Score maximum likelihood repeated-measures vs. placebo: ABT-126 25 mg: −2.13 at week 4 (90% confidence interval [CI] −3.36 to −0.89, *p* = 0.002), −2.06 at week 8 (90% CI −3.69 to −0.44, *p* = 0.018) and −1.61 at week 12 (90% CI −3.38 to 0.17, *p*= 0.068)
Gault et al., 2016 [46] (NCT01676935) Phase IIb	Randomized, double-blind, placebo-controlled trial	438 subjects with probable AD: (1) Placebo: 104 age 73.2 ± 7.39 (2) ABT-126 25 mg: 77 age 73.0 ± 7.62 (3) ABT-126 50 mg: 107 age 73.9 ± 8.26 (4) ABT-126 75 mg: 73 age 76.2± 8.14 (5) Donepezil 10 mg: 75 age 75.1 ± 7.75	NINCDS/ADRDA, MMSE 10–14, CSDD ≤ 10, MHIS ≤ 4	ADAS-Cog, AEs and safety	24 weeks	(1) Placebo (2) ABT-126 25 mg (3) ABT-126 50 mg (4) ABT-126 75 mg (5) Donepezil 10 mg	(1) 56 (2) 42 (3) 62 (4) 38 (5) 47	Total of 35	CIBICPlus vs. placebo at week 24 ABT-126 75 mg: −0.38 ± 0.13 (*p* = 0.002) ADSC-ADL vs. placebo at week 24 ABT-126 50 mg: 2.30 ± 1.04 (*p* = 0.013)
Frolich et al., 2011 [47] NCT00501111 Phase IIb	Multi-center, double-blind, double-dummy, randomized, placebo-controlled, parallel- group trial	Patients with probable AD: (1) Placebo: 164 age 73.5 ± 6.43 (2) AZD3480 5 mg: 80 age 74.0 ± 6.01 (3) AZD3480 20 mg: 80 age 73.8 ± 6.51 (4) AZD3480 35/100 mg: 84 age 72.7 ± 6.24 (5) Donepezil 5/10 mg: 161 age 73.9 ± 6.48	NINCDS-ADRDA, MMSE 12–26	ADAS-Cog, ADCS-CGIC, MMSE, DAD	12 weeks	(1) Placebo (2) AZD3480 5 mg (3) AZD3480 20 mg (4) AZD3480 35/100 mg (5) Donepezil 5/10 mg	(1) 60 (2) 21 (3) 23 (4) 34 (5) 60	(1) 6 (2) 7 (3) 7 (4) 19 (5) 22	Sub-analysis excluding very mild patients MMSE 25–26 resulted in slightly increased estimates of the effect size: −1.4, 95% CI: (−3.0; 0.2; *p* = 0.040) for AZD3480 20 mg. MMSE vs. placebo at week 12 AZD3480 20 mg: 0.8 ± 0.35 (*p* = 0.009) AZD3480 5 mg: 0.4 ± 0.35 (*p* = 0.091) ADCS-CGIC vs. placebo at week 12 AZD3480 20 mg: −0.5 ± 0.14 (*p* < 0.001) AZD3480 35/100 mg: −0.2 ± 0.14 (*p* = 0.070) Caregiver-reported outcomes DAD AZD3480 35/100 mg: 2.9 ± 2.15 (*p* = 0.090)
Pilot study									
Lynn Wilson et al., 1995 [48]	A double-blind, placebo-controlled trial	7 subjects (5 M, 2 W) with probable AD, age 78.6	DSM-III-R Guidelines, NINCDS- ADRDA Work Group, MMSE 14–22	Effect of sustained nicotine administration on behavior, cognition and physiology	22 days: 7 days placebo, 8 days nicotine, 7 days washout	(1) Placebo (2) Nicotine transdermal patch 22 mg	/	/	Learning errors intragroup nicotine: *p* < 0.05. On-task behavior intragroup nicotine increase of 14.0% (mean). Behavior observations intragroup nicotine 79.0% (mean)

Abbreviations: MCI: Mild Cognitive Impairment; AD: Alzheimer’s Disease; CSF: Cerebrospinal Fluid; CDR: Clinical Dementia Rating Scale; ADCS-ADL: Alzheimer’s Disease Cooperative Study—Activities of Daily Living Inventory; ADAS-Cog 70: Alzheimer’s Disease Assessment Scale—Cognitive Subscale 70; CGI-I: Clinical Global Impression—Improvement; NPI: Neuropsychiatric Inventory; CogState: Computerized Test Battery for Cognition; CPAL: Continuous Paired Associate Learning; MMSE: Mini-Mental State Examination; QoL-AD: Quality of Life in Alzheimer’s Disease; CIBIC-Plus: Clinician’s Interview-Based Impression of Change Plus; AE: Adverse Event; DEMQOL: Dementia Quality of Life; PPQSA: Partner-Patient Questionnaire for Shared Activities; WMS-III: Wechsler Memory Scale-III; CSDD: The Cornell Scale for Depression in Dementia; DAD: Disability Assessment for Dementia; ADAS-Cog-11: the 11-item Alzheimer’s Disease Assessment Scale-Cognitive subscale; ADCD-CGIC: Alzheimer’s Disease Cooperative Study—Clinical Global Impression of Change; SIB: Severe Impairment Battery; ADCS-ADLsev: Alzheimer’s Disease Cooperative Study—Activities of Daily Living (Severe); iADRS: Integrated Alzheimer’s Disease Rating Scale; RAVLT: Reay Auditory Verbal Learning Test; RVIP: Rapid Visual Intervention Processing; DRMLO: Delayed Response Matching to Location Order Task; IADL: Instrumental Activity of Daily Living; PDS: Progressive Deterioration Scale; SRT: Selective Reminding Task; RAT: Repeated Acquisition Test; SMT: Spatial Memory Task; CPT: Continuous Performance Task; SAV: Subjective Visual Analog; CIBIS: Clinician Interview-Based Impression of Severity; EQ-5D-5L: Quality of Life Using EuroQol-5 Dimension—Level 5; CMAI: Cohen-Mansfield Agitation Inventory; AAIQoL: Activity and Affect Indicators of Quality of Life; ZBI: Zarit Burden Interview.

**Table 3:** Synopsis of the mechanisms of action and therapeutic effects of the major nAChR agonists, as reported in published clinical trials. Abbreviations: MCI: Mild Cognitive Impairment; MMSE: Mini-Mental State Examination; ADCD-CGIC: Alzheimer’s Disease Cooperative Study—Clinical Global Impression of Change; BID: Bis In Die.

Molecule	Mechanism of Action	Therapeutic Effect Summary
Nicotine	- nAChrs agonist - stimulation of nAChRs in the central nervous system, which regulates the release of various neurotransmitters, such as dopamine, glutamate, serotonin, norepinephrine and γ-aminobutyric acid	In mild-to-moderate AD, the trials show a small effect on attention. However, no improvement was reported on memory, behavior and global cognition. Preliminary evidence shows a potential effect of nicotine on cognitive functions in people with MCI.
ABT-089	- selective neuronal nicotinic receptor (NNR) modulator - acts as an α4β2-nAChR partial agonist to stimulate the release of [3H]-dopamine	No improvement reported in mild-to-moderate AD.
AZD3480	- selective neuronal nicotinic receptor modulator - as an agonist, it has greater affinity for the α4β2 receptor than for the α7 receptor	One trial failed to show efficacy in reducing cognitive decline. However, some effect was reported in secondary outcomes (MMSE, ADCS-CGIC).
Varenicline	- nicotine bioisostere - stimulates the release of dopamine and reduces the self-administration of nicotine. Additionally, due to its characteristics, it is used as a pharmacotherapy for smoking cessation.	In one study, a single dose (1 mg BID for 4 weeks) showed no effect on memory, behavior and global cognition.
ABT-126	- partial agonist of α7 nAChR - has been shown to exhibit a maximum agonist activity of 74% in the human form of the α7 nAChR receptor	Different drug doses (5, 10, 25, 50, 75 mg) showed no statistically significant ability to reduce cognitive decline in mild-to-moderate AD.
ABT-418	- nicotine bioisostere - has greater selectivity for the α4β2 nicotinic receptor subtype and produces some in vitro effects similar to those produced by nicotine	Some effect was reported in different verbal learning and retrieval scores (selective reminding task: total recall; selective reminding task: recall failures), but the effect in several areas of memory impairment in dementia has yet to be tested.
PTI-125	- binds the scaffolding protein filamin A - reduces Aβ1-42’s binding affinity for a7nAChR, thereby preventing Aβ1-42’s signaling and further accumulation on a7nAChRs. It can prevent and reverse the binding of Aβ1-42 to α7nAChR.	An improvement in some biomarkers (total tau, p-tau181, neurofilament light chain, neurogranin, YKL-40, IL-6, IL-1β and TNFα) associated with AD was reported. The effects on cognitive decline are undergoing assessment, although the preliminary results seem encouraging.
